# A novel post-weld treatment using nanostructured metallic multilayer for superior fatigue strength

**DOI:** 10.1038/s41598-023-49192-0

**Published:** 2023-12-14

**Authors:** Jakob Brunow, Niclas Spalek, Fawad Mohammadi, Marcus Rutner

**Affiliations:** 1 Institute for Metal and Composite Structures, Hamburg University of Technology, Denickestr. 17, 21073 Hamburg, Germany; 2 Jörss-Blunck-Ordemann GmbH, Kaiser-Wilhelm-Str. 50, 20355 Hamburg, Germany

**Keywords:** Engineering, Civil engineering

## Abstract

Welded joints exhibit fatigue failure potential from weld geometry and characteristics of the heat affected zone. In order to counteract fatigue, structures and components require larger thicknesses resulting in heavier designs exhausting the finite natural resources. We hereby introduce a novel post-weld treatment, which postpones or even prevents fatigue failure of the welded connection. A Cu/Ni nanostructured metallic multilayer (NMM) is applied via electrodeposition and a 300–600% increase in usable lifetime compared to the untreated weld is observed. A FAT class 190 with a slope of k = 6 is proposed for the design of NMM treated butt welds. Material mechanisms responsible for the fatigue strength increase are introduced herein. A case study shows that the design of offshore wind turbine support structures applying NMM post-weld treatment enables a lifetime extension as well as a 28% weight reduction compared to the structure without post-weld treatment.

## Introduction

The pressing need to counteract environmental pollution and climate change has prompted research in civil engineering to explore ways to reduce its carbon footprint and adopt more environmentally friendly practices. One frontier in civil engineering is to extend the lifetimes of structures and reduce building material in the process, with the utilization of new and improved materials and construction techniques. In civil engineering, alongside corrosion, fatigue is responsible for premature failure of structures, extensive maintenance costs and safety risks. Particularly prone to fatigue loading is the welded connection, which is used in more than half of all global engineering products^1–6^. In order to extend the possible lifetime of welded connections post-weld treatments have been developed^7,8^. According to the International Institute of Welding (IIW) recommendations, post-weld treatment can be categorized into three groups. One focusses on methods to change the weld profile, e.g. grinding. The second group modifies the residual stress states in the weld proximity, e.g. High Frequency Mechanical Impact (HFMI) treatment, and the third improves the environmental conditions, e.g. resin coating^5,8,9^.

The nanostructured metallic multilayer (NMM) is applied locally on the weld and weld proximity covering the fatigue-susceptible area. NMM are known to possess superior material properties compared to the respective bulk materials and can be tailored towards special requirements^10,11^. This applies to corrosion resistance, strength, further, to magnetism, conductivity and radiation damage tolerance^12–22^. The novelty herein arises from using NMM as a supportive coating counteracting the fatigue susceptibility of welded connections. Increased fatigue life in NMM thin films has been shown in the literature. Especially Cu/Ni based systems show promising findings for the use as a post-weld treatment^11,18,22–34^. Additional factors for the metal selection include the cost advantage over precious metal constituents and the possibility for using electrodeposition as a rapid and scalable application method in contrast to physical vapor deposition or chemical vapor deposition^35^. The superior mechanical properties of NMM originate from mechanisms such as the Hall–Petch effect^36–38^ and are strongly dependent on the individual layer thickness^39–42^.

One example of a steel structure subjected to cyclic loading are support structures of offshore wind turbines, where fatigue is caused by water waves, wind and blade rotation^43^. The most common foundation used is the monopile, which is addressed in this study. Monopiles are being built in wind parks around the world to secure sustainable energy production. The design of monopiles is highly governed by fatigue. Wall thicknesses of up to 150 mm are necessary because of fatigue criticality of the circumferential welds which hold the monopile segments together. Fatigue limits the lifetime of the support structure to approximately 25 years^43^. Decommission of monopiles and re-erection are detrimental to the sea flora and fauna. Without the fatigue considerations in the design process, the monopile could be designed substantially thinner, which is investigated herein. Thereby leading to beneficial implications for the carbon footprint, the manufacturing process and the service lifetime of offshore wind turbines.

## Materials and experimental test setup

**Figure 1:**
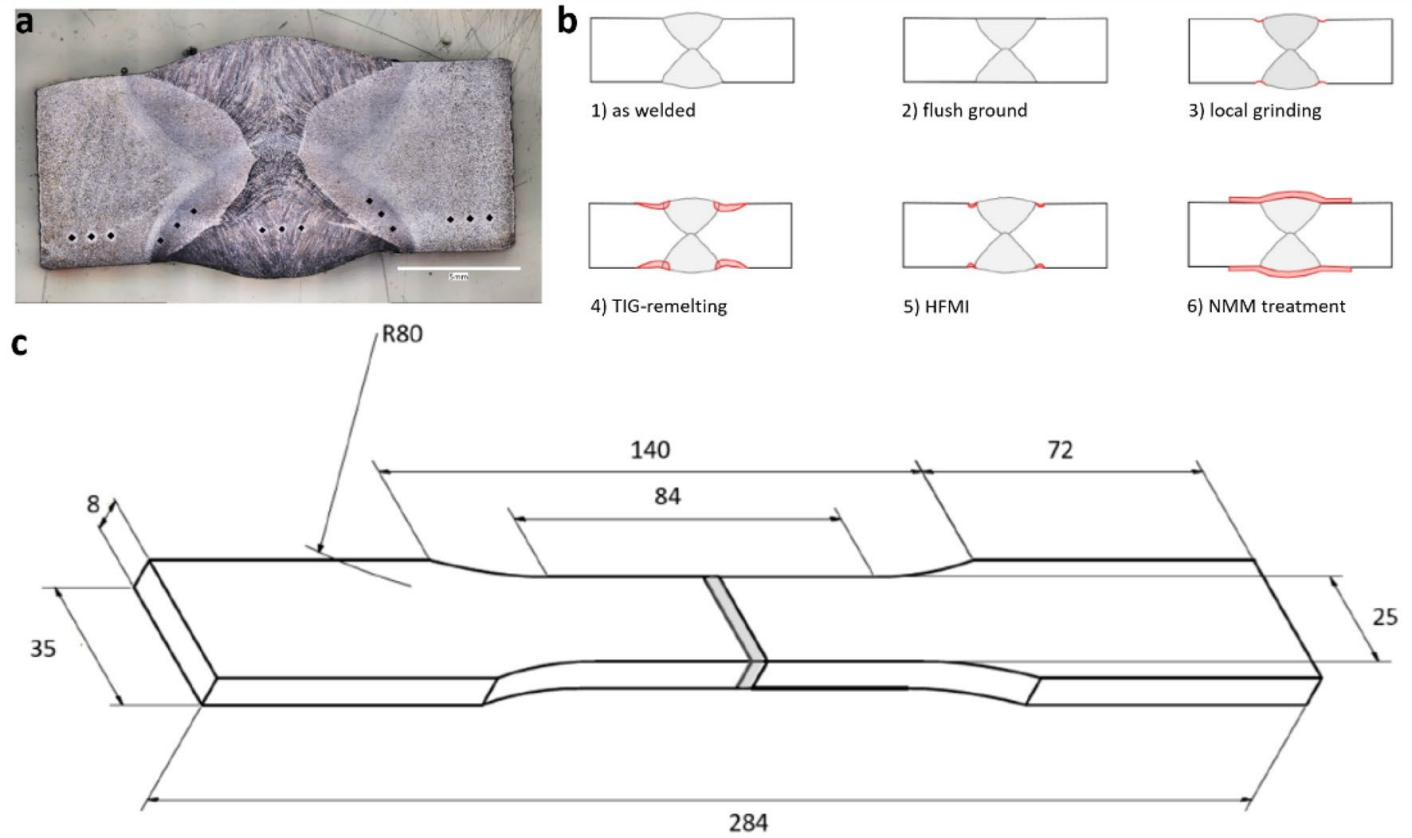
(**a**) Etched micrograph of the weld; (**b)** Schematic depiction of post-weld treatment methods for the double-sided V-butt weld; (1) As-welded cross-section of a double-sided V-butt weld; (2) Flush ground surface; (3) Local grinding of the weld toe; (4) TIG-remelted weld toes; (5) HFMI-treated weld toes; (6) NMM treated weld; (**c**) Dimensions of the dogbone specimen according to DIN 50125 Type E with double-sided V-weld.

In the research presented herein, the possibility of Cu/Ni nanostructured metallic multilayer (NMM) treatment for strengthening of welds is explored. Due to a profile change at the weld toe and a property change in the heat affected zone (HAZ), welds are vulnerable when subjected to cyclic loading. An etched micrograph of a weld, which is used for hardness measurement, is shown in Fig. 1a. Hardness values in the HAZ and the base material are 250 HV10 and 180 HV10, respectively. The nanostructured metallic multilayer treatment is schematically shown together with other post-weld treatments in Fig. 1b.

In Fig. 1c, the S355 J2 steel type-E specimen, as defined in DIN 50125^44^, with a center double V-weld, is shown. The height of the weld reinforcement is 1.3 ± 0.1 mm. The width of the weld face is 13 mm. Therefore, the weld of this study is categorized into FAT class 80 according to DIN EN 1993-1-9^3^. Table 1 lists the chemical compositions of base and filler materials.

**Table 1:** Chemical composition of base steel and filler material.

Material	C (wt%)	Mn (wt%)	Si (wt%)	*P* (wt%)	S (wt%)	Cu (wt%)
S355 J2	0.2	1.60	0.55	≤ 0.025	≤ 0.025	0.55
G3Si1	0.1	1.45	0.85	0	0	0

**Figure 2:**
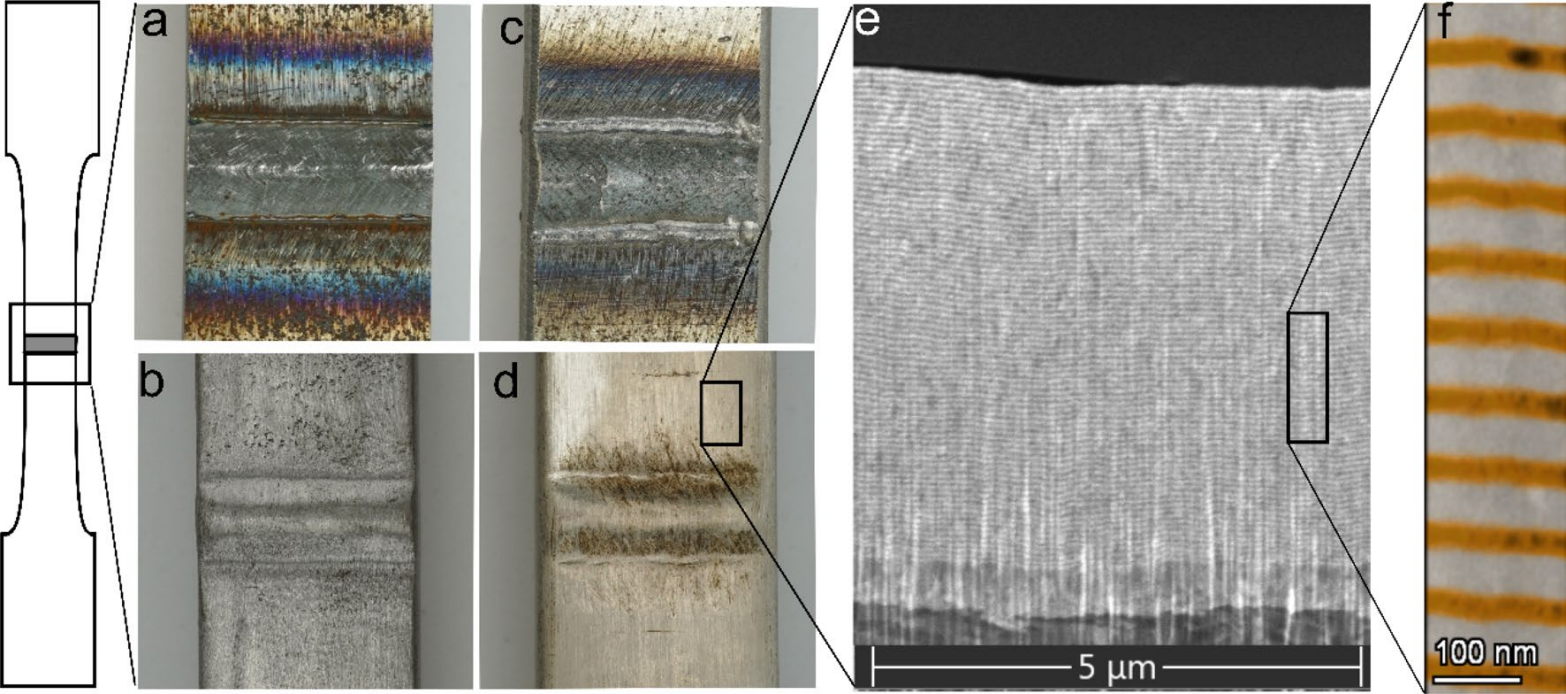
Micrographs of weld periphery: (**a**) As-welded specimen; (**b**) Polished specimen; (**c**) HFMI post-weld treated specimen; (**d**) NNM post-weld treated specimen; (**e**) SEM image showing the deposited NNM coating: The steel substrate (dark grey), the 1 µm-thick Ni base layer (light grey) and the Cu/Ni nanolaminate; (**f**) Close up colorized EDX/TEM image showing the sharp interfaces between Cu (orange) and Ni (grey) layers.

The NMM post-weld treatment is produced by electrodeposition^45–48^. The NMM metal combinations are limited by the available and stable electrolytes for the electrodeposition process^49^. The bilayers, consisting of almost pure individual Copper and Nickel layers, are deposited in a single-bath electrodeposition process. The current densities applied for the deposition of the Cu/Ni nanolaminate are 0.45 mA/cm^2^ for Cu deposition and 22 mA/cm^2^ for Ni deposition. The electrolyte consists of a citrate Cu/Ni sulfate bath^27^. The as-welded sample and the polished sample, further, the sample after HFMI post-weld treatment and the NMM post-weld treated sample are shown in Fig. 2a–d, respectively. The HFMI post-weld treatment is conducted by HiFIT Vertriebs GmbH, Aitrang, Germany. A 3 mm-diameter pin is used at 7–8 bar with a pin movement speed of 2.5 mm/s and a penetration depth of 0.15–0.25 mm. A SEM (FEI Helios NanoLAB G3) image of the Cu/Ni nanolaminate cross section with a Ni base layer deposited on the steel substrate is shown in Fig. 2e. An EDX/TEM (FEI Talos F200x) image, that distinguishes between the Cu (orange) and Ni (gray) layers, is depicted in Fig. 2f. The NMM lay-up has a 1,000 nm-thick Ni base layer and 160 Cu/Ni bilayers with a thickness of 50 nm each. Each bilayer consists of a 15 nm-Cu and a 35 nm-Ni thick layer.

## Fatigue testing and evaluation

Fatigue testing is performed with a servo-hydraulic uni-axial testing machine (Schenck PC400M) and evaluated according to DIN 50100^50^ and recommendations of DVS notice 2403^51^. The constant amplitude loading is a sinusoidal load with a static mean stress S_m_, the stress amplitude S_a_, whereas a wide range of stress is covered to generate the stress (S)—cycle number (N) curve. The stress ratio of all tests is R = 0. All fatigue tests are conducted with a frequency of 8 Hz. The samples are tested until fracture or until cycle number N = 2.5*∙*10^6^ is reached, which is defined as run-out. Table 2 summarizes the sample test matrix and provides the sample definition, the number of specimen tested and the slope of the S–N curve assessed.

**Table 2:** Summary of fatigue test data.

Category of specimen	Number of specimen	Number of run-outs	Slope k of S–N curve assessed according to DIN 50100^50^	Modified slope k according to DIN 50100^50^
As-welded	32	3	2.57	3
HiFIT	20	2	3.46	5
Polished	11	4	3.2	3
NMM	16	4	6.79	6

**Figure 3:**
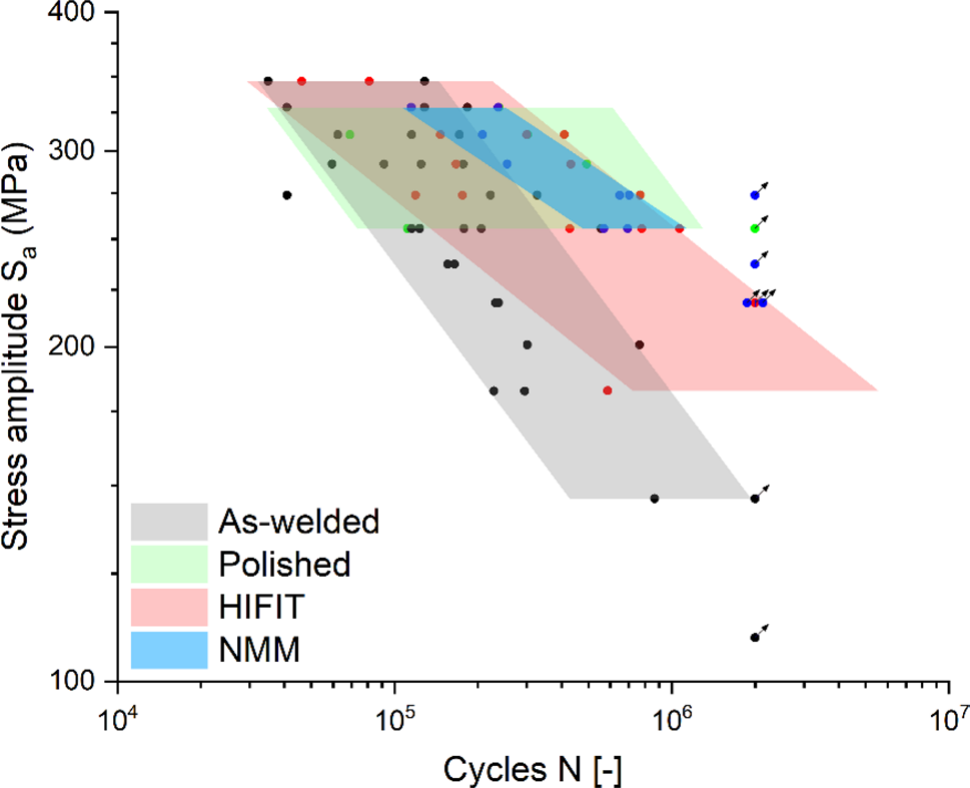
Scatter plot of fatigue specimen with colored areas showing the 80%-confidence interval, bound by the 10%- and the 90%-quantile; Comparative study of as-welded, polished, HFMI (HiFIT) post-weld treated and NMM post-weld treated specimen.

Figure 3 shows all measured data points with shaded areas indicating the 80%-confidence intervals of the different data sets. This plot demonstrates that the scatter of the as-welded specimen is relatively high. The scatter even increases after polishing of the surface. The HFMI post-weld treatment (HiFIT) achieves a reduced slope of the S–N curve, however, a significant scatter remains. In contrast, the NMM treated specimen reveal a very narrow distribution of data points.

In engineering design terms, a reduced scatter equates to a high level of effectiveness and reliability of the technology. Obviously, these findings provide a first strong indication, while further testing and enlarging the data set is necessary to confirm this.

**Figure 4:**
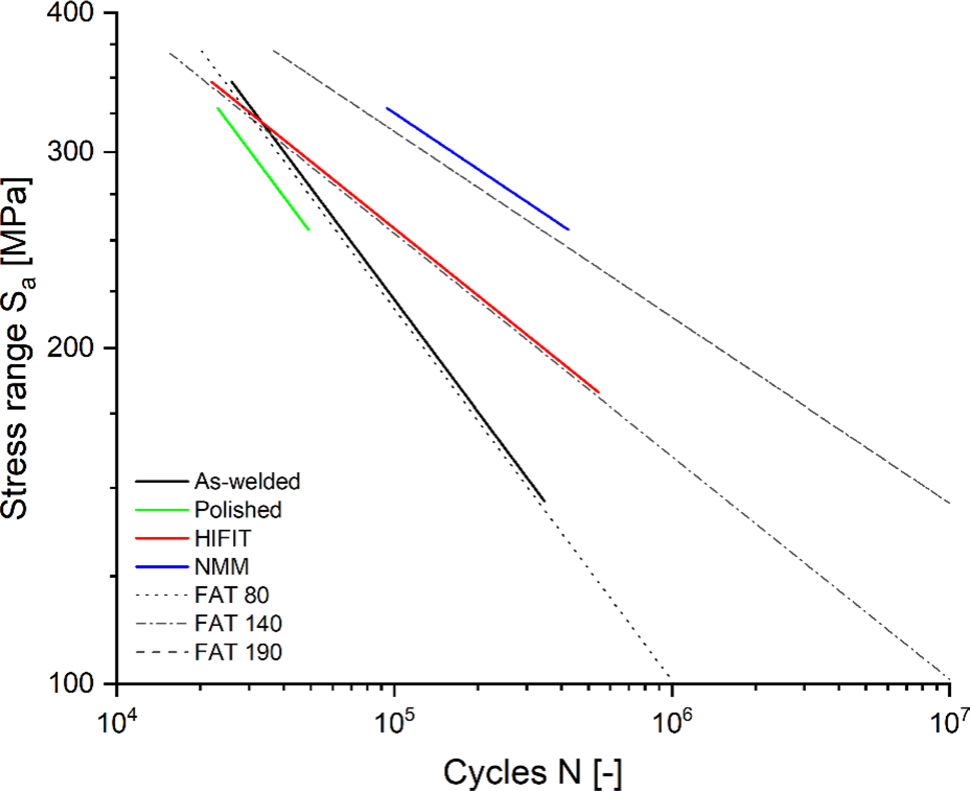
Design S–N curves derived from the 5%-quantiles compared to the corresponding FAT classes.

Figure 3 shows the 80% confidence intervals bound by the 10% and 90% quantiles, however, the design of structures according to Eurocode^3^ is based on the 5% quantile, as plotted for all categories of samples in Fig. 4. The plot includes the corresponding FAT classes from the literature. It should be noted that the 5% quantile of the as-welded specimen is almost congruent with the FAT class 80 which is in agreement with Eurocode^3^. Further, the 5% quantile of the HFMI (HiFIT) post-weld treated specimen is almost lining up with FAT class 140 with a slope of k = 5, which is consistent with the fatigue class for HFMI used in the literature^52^. The experimental data matching the corresponding FAT classes according to specifications can be interpreted as a validation of test setup and test performance. The 5% quantile of the polished specimen shows no increase in fatigue life resulting from the large scatter. Further, a lifetime improvement for Cu/Ni NMM treated welds is seen across all stress ranges. The 5% quantile of the Cu/Ni NMM treated specimen achieves a FAT class 190 with k = 6, which corresponds to a fatigue design lifetime increase between 300 and 600% depending on the stress range. NMM treated specimen show run-outs below a stress range of 70% of yield strength which equates to 249 MPa.

## Fundamental effects of NMM

**Figure 5:**
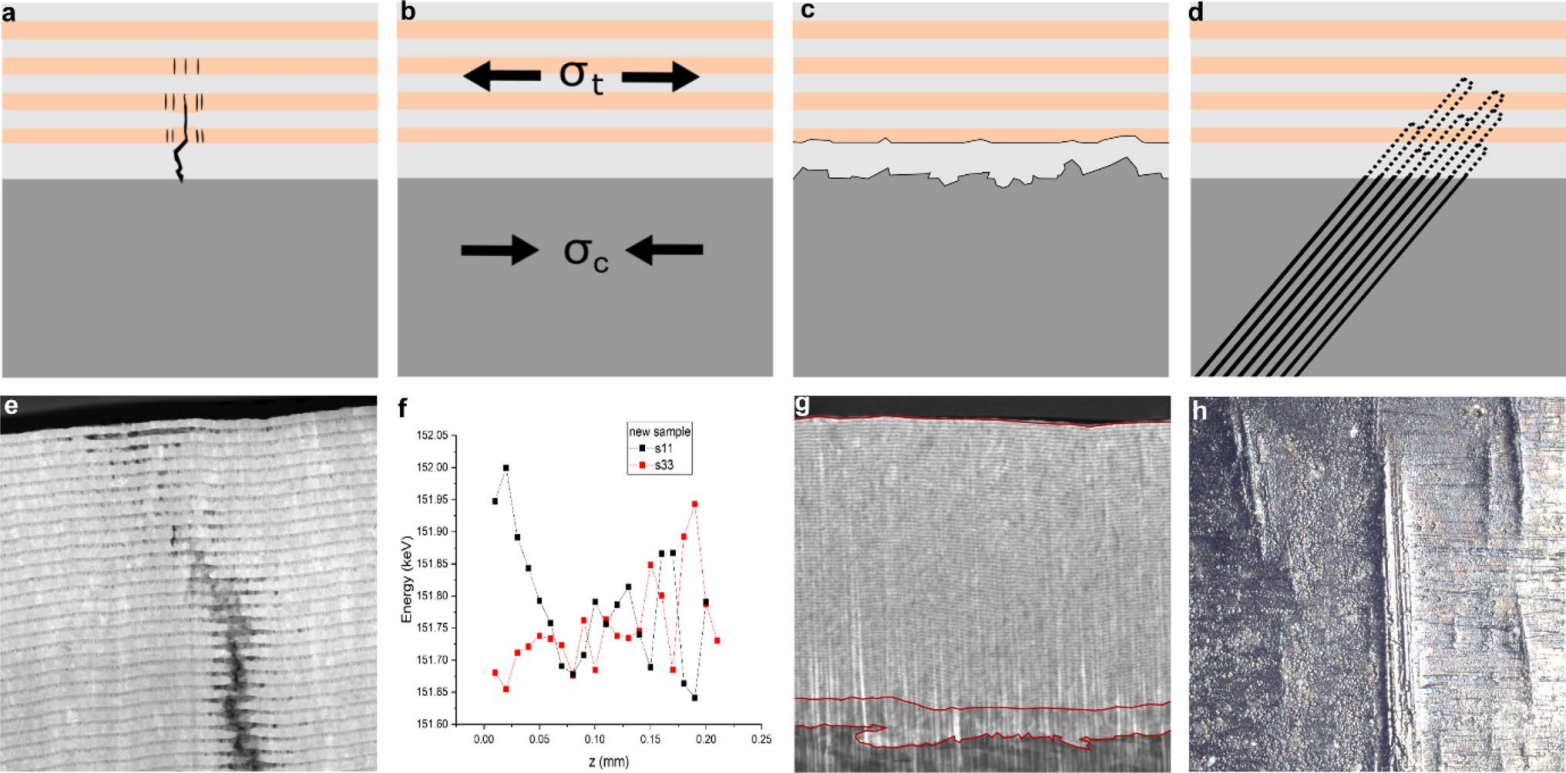
Schematic depictions of underlying principles of improved fatigue resistance: (**a**) Influenced crack propagation through NMM; (**b**) Introduction of residual tensile stresses in the NMM and equivalent compressive stresses in the steel substrate adjacent to the surface; (**c**) Reduction of surface roughness; (**d**): Suppression of PSB by hard coating application; (**e**) TEM image of complex crack propagation across NMM; (**f**) Synchrotron energy data revealing residual compressive stress gradient in steel surface proximity after NMM application; (**g**) SEM image with visualized surface roughness reduction; (**h**) Image of visible extrusions in HAZ after cyclic loading.

Results from FIB-SEM tomography and subsequent TEM investigations indicate, that crack propagation through NMM is accompanied by multi crack formation within the adjacent Cu layers, as well as necking and work hardening in the Ni layers^28,53–56^, as shown in Fig. 5a. The schematic sketch shows the dark grey steel substrate, the light grey Ni base layer and the Cu/Ni nanolaminate. Complex crack dynamics, including deflection, arrest, crack re-initiation and aforementioned multi-crack development and work hardening are responsible for energy dissipation in the crack propagation phase, as shown in Fig. 5e. This is seen as one explanation for the observed superior fatigue resistance^28^. While this *crack propagation* behavior is in line with common explanations for the increased strength of NMM and the observations are well supported by findings from the literature^28^, the following mechanisms are expected to significantly contribute to an increase in fatigue strength through a postponed *crack initiation.* Further research is needed to quantify the individual contribution of the following expected mechanisms:

- Compressive residual stresses introduced to the steel surface (Fig. 5b)
- Surface roughness reduction (Fig. 5c)
- Suppression of persistent slip bands (PSB) (Fig. 5d)

A preliminary synchrotron radiation X-Ray diffraction (XRD) experiment indicates a complex residual stress behavior. Significant tensile residual stress of several hundred MPa is measured in the nanolaminate while compressive stress is measured in the steel substrate adjacent to the surface (Fig. 5b). A gradient of residual stress over the thickness of the cross section is observed in measurements conducted at PETRA-III at DESY German Electron Synchrotron in Hamburg, Germany, as shown in Fig. 5f. The compressive residual stress in the steel substrate is assumed to compress microcracks, hence, contributing to postpone or even avoid crack initiation. The residual stress states are measured in the nanolaminate of the unloaded specimen and after fatigue failure. It is recognized that the tensile stress in the Ni layers even increases throughout the lifetime which can be attributed to the load transfer from Cu layers to its neighboring Ni layers due to multi-cracking.

The surface roughness is reduced through the deposition of the NMM, as shown in Fig. 5c,g. Surface defects as potential sources for fatigue crack initiation are mitigated. Red lines sketched into Fig. 5g show the surface roughness of the polished steel substrate compared to the interface between Ni base layer and nanolaminate and on top of the nanolaminate. As recently shown, the surface roughness at the end of lifetime for the NMM treated weld is smoother than the roughness of the pristine polished steel^28^.

PSB form in surface proximity and become detectable by intrusions and extrusions during cyclic loading (Fig. 5h). Intrusions are widely considered to be the nucleation point for crack formation. Recent simulated investigations of the interaction between PSB and surface hard coatings demonstrate that the suppression of PSB increases the fatigue lifetime of materials^31,57,58^. Further investigations are necessary to qualitatively describe and to quantify the influence of PSB suppression by NNM coatings to prolong the crack initiation phase (Fig. 5d).

Summarizing, NMM treatment addresses all three main improvement techniques defined by IIW^5^, notably the improvement of weld profile, residual stress conditions as well as environmental conditions.

## Lifetime extension and steel mass savings of wind turbine structures

The steadily increasing targets for offshore wind energy production^59^ pose a significant challenge to the supply chain to meet the future demand. In particular, the monopile structure as the most common form of foundation^60^ and heaviest component is prone to fatigue failure and poses a significant challenge for production and handling.

**Figure 6:**
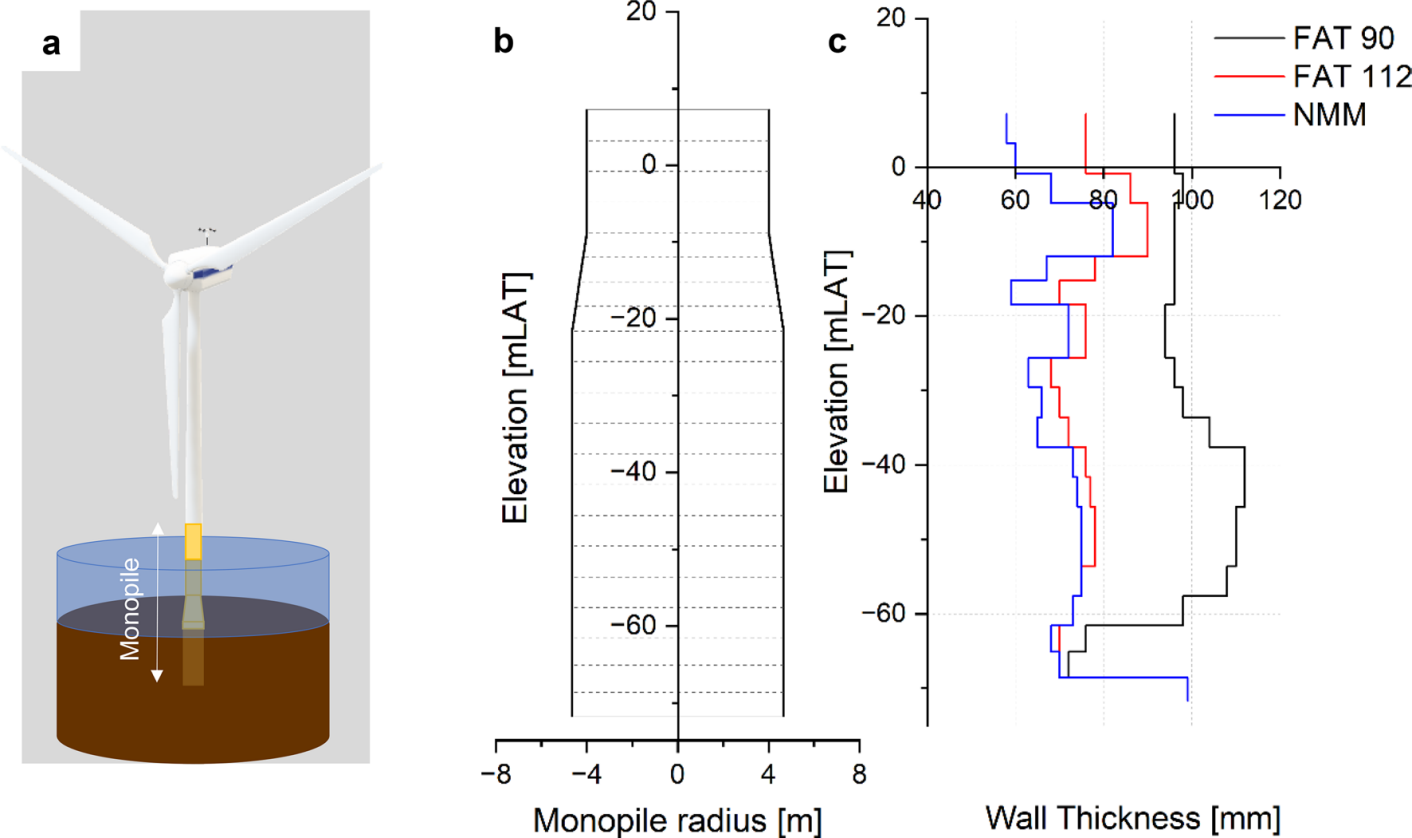
(**a**) Offshore wind turbine structure; (**b**) Schematic depiction of a monopile foundation. Circumferential welds represented by dotted lines; (**c**) Resulting wall thickness distribution for the respective configurations A–C.

The case study determines what effect the NMM treatment has, if applied on the circular welds of a monopole type foundation with a state-of-the-art 15 MW offshore wind turbine (Fig. 6a). The fully integrated numeric model consists of the rotor-nacelle-assembly, tower, substructure and foundation and is analyzed in a hydrodynamic and aeroelastic finite-element analysis using the software *Bladed*^61^. The environmental boundary conditions regarding water depth, geotechnical resistance, metocean data are selected to resemble a typical location in the German North Sea.

The analysis covers a wide range of load cases:

- Fatigue limit state (FLS), e.g. operational load cases during power production
- Ultimate limit state (ULS), e.g. an extreme weather event
- Service limit state (SLS), e.g. a severe weather event with a focus on deflections
- Installation load cases, e.g. loads during pile driving

The codes of the International Electrotechnical Commission (IEC)^62^ specify the number of categories of load cases that need to be analyzed for the design of foundations of offshore wind structures.

The most critical load cases drive the design of the monopile. Typically, the ULS and SLS load cases drive the embedment depth and bottom outer diameter of the monopile, FLS usually—and in certain cases ULS—drives the distribution of wall thickness over height. Further, minimizing the top diameter leads to overall smaller loads as the surface area subjected to wave forces is minimized. The starting configuration of the case study contains an optimized monopile design based on the normative fatigue resistances without consideration of the NMM treatment. In this case study several configurations are simulated. Global parameters such as the outer monopile diameter and embedment depth are not changed among the configurations as a change of these parameters has a significant influence on the applied load level. For every reduction in wall thickness, as shown in Fig. 6c, a structural reassessment is performed to determine the applicable loads based on the changes in stiffness. The analyses revealed that although the thickness reductions are substantial, changes in eigenfrequency and load level are minor.

The following three configurations are distinguished:

*Configuration A* Resembling the current design approach. The majority of the circumferential welds (Fig. 6b) connecting the segments of the monopile remains in the as-welded condition (FAT 90) with the exception of the welds below and above the conical transition piece to be improved via flush-grinding (FAT 112). The total weight sums up to 1710 t.

*Configuration B* All welds of the monopile (Fig. 6b) are flush ground (FAT 112) representing the theoretical optimum in respect to fatigue resistance according to DNV^63^. The total weight sums up to 1337 t, which is a 22% weight reduction compared to configuration A.

*Configuration C* Covering the monopile with NMM post-weld treatment applied on the circumferential welds (Fig. 6b). As the NMM by far extends the fatigue resistance of the base metal, the applicable fatigue resistance is set to FAT 140 within the case study, which resembles the fatigue resistance of the base metal. The total weight sums up to 1236 t, which is a 28% weight reduction compared to configuration A.

The results of configuration A confirm that fatigue susceptibility of the untreated welds drives the design of monopiles^43^. Post-weld treatments reduce the fatigue criticality. Configuration B, although considered to be economically challenging, demonstrates, that a significant wall thickness reduction is achievable, as shown in Fig. 6c. However, the design in configuration B remains mostly driven by the FLS. For configuration C, the advantages of a NMM post-weld treatment cannot be fully utilized since further reduction of the wall thickness is restricted by ULS-criteria, such as buckling and plastic deformation. However, the NMM treatment eliminates the fatigue criticality which allows an 300–600% extended service life and at the same time enables a 8% further weight reduction compared to configuration B.

By neutralizing the fatigue susceptibility of welded structures, the NMM enables the offshore wind industry to further utilize their current production capacities. The application of the NMM post-weld treatment further contributes to the global energy production targets, sustainable use of current infrastructure and efforts to minimize green-house gas emissions.

## Summary

Fatigue tests of welded S355 J2 steel type-E specimen according to DIN 50125^44^, comparing the NMM treated welds to untreated and conventionally post-weld treated (HFMI) welds, reveal a superior fatigue strength. The S–N curve of the NMM treated weld corresponds to a FAT class 190. Four underlying material mechanisms, which are seen as responsible for the significant fatigue strength increase, are discussed. An additional case study investigates NMM treatment of all circumferential welds of a 15 MW reference wind turbine monopile foundation, assessed in a hydrodynamic and aeroelastic finite element analysis. Complete elimination of the fatigue criticality of all welds and additionally a 28% weight reduction of the structure is identified.

With these findings, a novel post-weld treatment method is herein introduced with the potential to change the state-of-the-art in design and maintenance of welded structures, hence contributing to sustainable use of infrastructure and natural resources and reduction of the carbon footprint of the steel industry.

## Methods

### Sample preparation

S355 J2 steel type-E specimen, as defined in DIN 50125^44^, with a center double V-weld, are used. The weld material is G3Si1 (Table 1). NMM post-weld treatment is produced by electrodeposition. The NMM lay-up has a 1000 nm-thick Ni base layer and 160 Cu/Ni bilayers with a thickness of 50 nm each. Each bilayer consists of a 15 nm-Cu and a 35 nm-Ni thick layer. The bilayers are deposited in a single-bath electrodeposition process. The current densities applied for the deposition of the Cu/Ni nanolaminate are 0.45 mA/cm^2^ for Cu deposition and 22 mA/cm^2^ for Ni deposition. The electrolyte consists of a citrate Cu/Ni sulfate bath. The high frequency impact post-weld treatment is conducted using a 3 mm-diameter pin at 7–8 bar with a pin movement speed of 2.5 mm/s and a penetration depth of 0.15–0.25 mm.

### Synchrotron radiation X-ray diffraction (XRD)

The X-Ray diffraction tests are performed with the high energy beamline P61A at PETRA-III (DESY synchrotron facility Hamburg, Germany). The beamline is optimized for energy dispersive measurements with usable photon energies ranging from 30 to 200 keV. The experiments are conducted in transmission mode using a HPGe point detector, with *2ϴ* ~ 4°. Sample positioning is done using an Eulerian cradle. Gauge volumes of 10 × 500 × 500 µm^3^ and 100 × 10 × 10,000 µm^3^ for the respective stresses s33 and s11 are set up in the HAZ with the scan direction being vertical (in the direction of the cross section).

### Fatigue testing

Fatigue testing is performed with a servo-hydraulic uni-axial testing machine (Schenck PC400M). The constant amplitude loading is a sinusoidal load with a static mean stress S_m_, the stress amplitude S_a_, whereas a wide range of stress is covered to generate the stress (S)—cycle number (N) curve. The stress ratio of all tests is R = 0. All fatigue tests are conducted with a frequency of 8 Hz. The samples are tested until fracture or until cycle number N = 2.5 × 10^6^ is reached, which is defined as run-out. The S–N curve is established according to DIN 50100^50^*.*

### Finite element analysis

A fully integrated model consisting of turbine, tower, substructure and foundation is modelled in a hydrodynamic and aeroelastic finite element analysis using the software Bladed^61^.

## Data Availability

The data presented in this study are measured data by Synchrotron radiation X-Ray diffraction (XRD), Electron microscopy and fatigue tests and are available on request from the corresponding author.
